# Avian malaria and bird humoral immune response

**DOI:** 10.1186/s12936-018-2219-3

**Published:** 2018-02-09

**Authors:** Jessica Delhaye, Tania Jenkins, Olivier Glaizot, Philippe Christe

**Affiliations:** 1 Department of Ecology and Evolution, University of Lausanne, Le Biophore, Unil Sorge, 1015 Lausanne, Switzerland; 2 Museum of Zoology, Place de la Riponne 6, 1005 Lausanne, Switzerland

**Keywords:** Keyhole limpet haemocyanin, *Plasmodium relictum*, *Serinus canaria*

## Abstract

**Background:**

*Plasmodium* parasites are known to impose fitness costs on their vertebrate hosts. Some of these costs are due to the activation of the immune response, which may divert resources away from self-maintenance. *Plasmodium* parasites may also immuno-deplete their hosts. Thus, infected individuals may be less able to mount an immune response to a new pathogen than uninfected ones. However, this has been poorly investigated.

**Methods:**

The effect of *Plasmodium* infection on bird humoral immune response when encountering a novel antigen was tested. A laboratory experiment was conducted on canaries (*Serinus canaria*) experimentally infected with *Plasmodium relictum* (lineage SGS1) under controlled conditions. Birds were immune challenged with an intra-pectoral injection of a novel non-pathogenic antigen (keyhole limpet haemocyanin, KLH). One week later they were challenged again. The immune responses to the primary and to the secondary contacts were quantified as anti-KLH antibody production via enzyme-linked immunosorbent assay (ELISA).

**Results:**

There was no significant difference in antibody production between uninfected and *Plasmodium* infected birds at both primary and secondary contact. However, *Plasmodium* parasite intensity in the blood increased after the primary contact with the antigen.

**Conclusions:**

There was no effect of *Plasmodium* infection on the magnitude of the humoral immune response. However, there was a cost of mounting an immune response in infected individuals as parasitaemia increased after the immune challenge, suggesting a trade-off between current control of chronic *Plasmodium* infection and investment against a new immune challenge.

## Background

Avian malarial parasites can impose fitness costs on their vertebrate hosts and several studies have shown that infection can lead to decreased reproductive success [1], decreased survival [2] or increased telomere degradation [3]. Part of these costs may be mediated by the activation of the host immune response which may be engaged in several life history trade-offs with for example self-maintenance, reproduction and survival [4, 5].

Immune functions require energy. For example, immune activation has been shown to depend on energy storage [6, 7] and to affect host metabolism [7–10]. The immune response may also be harmful because of its inherent propensity to cause auto-immunity and oxidative stress, leading to host self-reactivity and damage to somatic cells [11–13]. An increased susceptibility to oxidative damage due to immune activation [14] has also been linked to reduced survival [15, 16]. Therefore, a trade-off may occur between mounting an immune response and self-maintenance.

Parasites have also evolved evasion strategies to avoid the host immune defence and to establish in their host [17–19]. For instance, malarial parasites have been suggested to adaptively immuno-deplete their human and mouse hosts by interfering with immune signalling [20–23]. Therefore, malarial parasites may impose an additional cost to their vertebrate host due to the depletion of immune functions which may increase host susceptibility to other parasites and diseases.

In the present study, the effect of *Plasmodium* infection on the ability of hosts to mount an immune response when encountering a new pathogen was investigated. A laboratory experiment was conducted under controlled conditions with experimental infections on non-breeding canaries (*Serinus canaria*). Bird humoral immune response was measured as the antibody production after injection with a novel non-pathogenic antigen. A novel antigen was used to test for the ability to mount an immune response without the problem of unknown past exposures and potential acquired immunity. *Plasmodium* infected individuals were predicted to have an impaired immune response when faced with a novel antigen. Alternatively, if their immune response was not impaired, they would pay a higher cost (in terms of higher anaemia or weight loss) of immune activation.

## Methods

### Laboratory experiment

To compare levels of humoral immune response of *Plasmodium* infected and uninfected birds under controlled conditions, canaries (*Serinus canaria*) were experimentally infected with *Plasmodium relictum* (lineage SGS1) in the laboratory. Thirty-two 1-year old canaries (15 females and 17 males) were kept in randomized groups of one to three individuals of the same sex per cage (1 × 1 × 2 m) in the animal facility (14:10 day–night light cycle, 20 °C, 55% humidity). Each of the 16 cages received fresh food (mix of 24 g of seeds, Vitabalance, and 18 g of couscous, Migros Bio) and fresh water (500 mL) daily, as well as a vitamin mix (Océvit, Virbac, 1 mL per litre of water) weekly. A first group of canaries (eight females and nine males) were experimentally infected via intra-peritoneal injection of 75 µL of a blood mix (prepared from blood of canaries previously infected with *P. relictum*) mixed with PBS (1:1). The control group (seven females and eight males) received an injection of the same volume of PBS. Once the infected canaries reached the chronic phase of infection (42 days post infection, hereby dpi, [24]), all the canaries were blood sampled and immune challenged (primary contact). At 49 dpi, they were blood sampled and immune challenged a second time (secondary contact). A final blood sample was taken at 56 dpi. Individuals were weighed prior to each blood sampling. The experiment was designed in two equivalent temporal blocks 1 week apart. After blood collection, haematocrit was measured in capillaries as the fraction of red blood cells in the total blood volume. The rest of the blood was centrifuged for 10 min at 4 °C at 15,000 relative centrifugal force. Plasma was stored at − 80 °C until immunological testing and red blood cells were stored at − 20 °C until *Plasmodium* detection and quantification.

Potential differences in food intake due to different energetic demands between uninfected and *Plasmodium* infected individuals were controlled by assessing individual daily food consumption. The daily food consumption per cage (in g/cage/day) was measured by weighing each morning the unconsumed amount of food and was used to extrapolate the average amount of food consumed per canary per day (in g/individual/day). For this reason, all the individuals of a cage received the same infection treatment.

### Immune challenge

In order to measure the bird humoral immune response when encountering a new pathogen, keyhole limpet haemocyanin (KLH), a molecule extracted from the giant keyhole limpet (*Megathura crenulata*) was used as an antigen. This molecule elicits a humoral immune response without pathogenic effects. A solution of KLH (Sigma-Aldrich) emulsified in PBS (1 mg/mL) and mixed with incomplete Freund’s adjuvant (Sigma-Aldrich, 1:1) was prepared according to [25]. Each bird received an intra-muscular (pectoral muscle) injection of 50 µL of KLH-adjuvant solution (25 µg of KLH per injection, adapted from [25]).

### Humoral immune response quantification

The humoral immune response when encountering a novel antigen (KLH) was measured by quantifying the anti-KLH antibodies present in the plasma via enzyme-linked immunosorbent assay (ELISA). Plates (Nunc, MaxiSorp, Thermo Fisher Scientific) were incubated over night at 4 °C on a turning plate with KLH solution (0.5 mg KLH per mL in sodium hydrogen carbonate 0.1 M pH 9.6). Coated plates were washed with PBS-Tween 20 0.05% (PBS-T) and incubated with PBS-T containing 5% of non-fat dry milk for 2 h at ambient temperature on a turning plate. Plates were washed with PBS-T and incubated with standards (eight standards prepared with immunoglobulin Y of chicken immunized against KLH, Gallus Immunotech Inc., concentrations from 20 μg/mL to 2 ng/mL diluted in PBS) and samples (before and after immune challenge, 1:100 dilution in PBS) all in duplicates for 3 h at 37 °C on a turning plate. Plates were washed with PBS-T and incubated with a secondary antibody (anti-chicken immunoglobulin Y peroxidase-linked antibodies produced in rabbit, Sigma-Aldrich, 1:1000 dilution in PBS) for 1 h at 37 °C on a turning plate. Plates were washed with PBS-T and incubated with *O*-phenylenediamine dihydrochloride peroxidase substrate (Sigma-Aldrich, 0.4 mg/mL) for 30 min in the dark at ambient temperature. The reaction was stopped by adding HCl 3 M and absorbance was read at 560 nm with a spectrophotometer. Anti-KLH antibody level was extrapolated from the standard curve and expressed in mg/mL. Negative controls (blank and non-specific binding) were performed in duplicates on each plate.

### *Plasmodium* detection and quantification

DNA was extracted from red blood cells using the DNeasy blood and tissue extraction kit (Qiagen) according to the manufacturer’s protocol for the BioSprint 96. A nested PCR was performed to detect the infection status of individuals. Following a primary reaction with the primers HaemNF1 and HaemNR3, a secondary reaction with the primers HaemF and HaemR2 to amplify *Plasmodium* was conducted as described in [26] adapted from [27] and 5 μL of PCR product was run on a 2% agarose gel to assess infection status.

Parasite quantification was performed by quantitative PCR as described in [26]. Briefly, two separate qPCR reactions using a parasite cyt b TaqMan probe (CY3-CYTb-BHQ2) and a host 18 s rRNA probe (FAM-18S-BHQ1) were performed. For both parasite and host, DNA concentration was calculated from the standard curve and the parasitaemia was given by the ratio of parasite DNA concentration on the host DNA concentration. In order to normalise the distribution, this ratio was log_10_ transformed. As a consequence, we obtained a range of parasitaemia values increasing from − 2.10 to 1.10 (arbitrary unit) in infected canaries.

### Statistical analyses

Statistical analyses were performed in R (version 3.1, [28]).

Anti-KLH antibody level was analysed as a response variable in linear mixed effect models (lme function in nlme package). Terms for sex (female–male), infection group (control–infected), antigen contact (primary–secondary contact) and all two-way interactions were included. Haematocrit and body mass were also analysed as response variables in linear mixed effect models including terms for sex (female–male), infection group (control–infected), antigen contact (prior to immune challenge—after primary contact—after secondary contact) and all two-way interactions. For *Plasmodium* infected canaries, parasitaemia over time was also investigated. Parasitaemia was analysed as a response variable in a linear mixed effect model including terms for sex, antigen contact (prior to immune challenge—after primary contact—after secondary contact) and their two-way interaction. Daily food consumption, calculated at the cage level, was analysed as a response variable in linear mixed effect models including terms for the density of canary per cage (1–3, to control for heterogeneity in the number of individuals per cage), sex (female–male), infection group (control–infected), time (as a quantitative continuous variable) and all two-way interactions between sex, infection group and time. Random factors were implemented as canary identity nested in cage nested in block (only cage nested in block for food consumption as it was calculated at the cage level) to account for the nested experimental design. An auto-correlation structure (corAR1) was implemented to account for repeated measurements. To determine the explanatory power of each fitted parameter, likelihood ratio tests were conducted following a standard backward selection procedure by sequential elimination of each fitted terms of similar order from the full model [29]. Only significant terms were kept to reach the minimal adequate model. The significant p values given in the text come from the minimal adequate models and the non-significant p values come from the likelihood ratio tests prior to the elimination of the non-significant term from the model. To look at the effect of each significant term individually, contrast analyses were performed [29].

As haematocrit is usually negatively affected by parasitaemia, the link between both parameters was also investigated. To account for the nested experimental design and repeated measurements, haematocrit was analysed as a response variable in a linear mixed effect model, implementing canary identity nested in cage nested in block as random factors and an auto-correlation structure (corAR1).

## Results

**Fig. 1:**
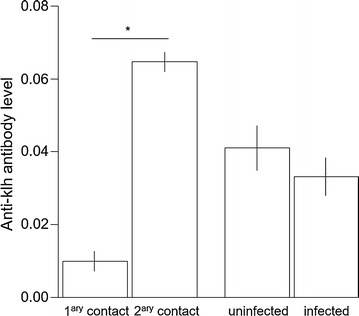
Humoral immune response after immune challenges. Anti-KLH antibody level (mg/mL) after primary and secondary contact to KLH antigen and in uninfected and infected individuals. The star indicates a significant difference

There were no anti-KLH antibodies in the plasma prior to the first KLH injection. Anti-KLH antibody production was elicited after the primary contact and significantly increased after the secondary contact (Table 1A, Fig. 1). There was no significant difference of anti-KLH antibody level between the infection groups or between the sexes (Table 1A).

**Table 1:** Summary table of linear mixed effect models

	Estimate	se	t value	*p* value
A—Anti-KLH antibody level				
Intercept	0.0135	0.0044	3.03	0.0049
Antigen contact	0.0546	0.0038	14.23	< 0.0001
Infection	− 0.0071	0.0035	− 2.03	0.0630
Sex				0.3852
Sex:infection				0.9385
Sex:antigen contact				0.6984
Infection:antigen contact				0.8441
B—Parasitaemia				
Intercept	− 1.3100	0.3637	− 3.60	0.0013
Primary contact	0.4480	0.1722	2.60	0.0149
Secondary contact	0.8352	0.2123	3.93	0.0005
Sex				0.7583
Sex:antigen contact				0.7733
C—Haematocrit				
Antigen contact				0.8830
Infection				0.3684
Sex				0.4051
Sex:infection				0.3616
Sex:antigen contact				0.8048
Infection:antigen contact				0.9807
D—Body mass				
Intercept	24.6219	0.4981	49.43	< 0.0001
Primary contact	− 0.3594	0.1226	− 2.93	0.0047
Secondary contact	− 0.2631	0.1290	− 2.04	0.0457
Infection				0.4846
Sex				0.5193
Sex:infection				0.6287
Sex:antigen contact				0.9930
Infection:antigen contact				0.3580
E—Daily food consumption				
Intercept	12.8814	1.1213	11.49	< 0.0001
Time	0.0438	0.0170	2.58	0.0106
Number of canaries per cage	− 3.2332	0.3168	− 10.21	< 0.0001
Sex				0.8609
Infection				0.8316
Sex:infection				0.2238
Sex:time				0.5214
Infection:time				0.7816

A: anti-KLH antibody level (32 canaries measured twice), B: parasitaemia (log transformed) in infected canaries (17 canaries measured 3 times), C: haematocrit (32 canaries measured 3 times), D: body mass (32 canaries measured 3 times) and E: daily food consumption per canary per cage (16 cages measured 14 times). Minimal models are given with intercept as well as estimates, standard errors (se), t values and p values for each specific term. Non-significant terms are given with the p value of the likelihood ratio test before being dropped-out of the model

**Fig. 2:**
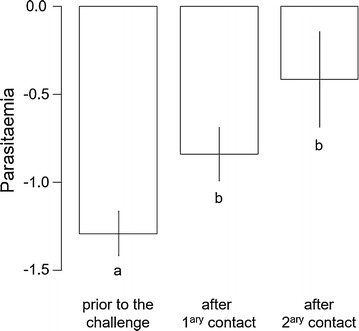
Parasitaemia prior to and after immune challenges. Parasitaemia (arbitrary unit, log transformed) as a function of antigen contact in infected canaries prior to the immune challenge, after primary contact and after secondary contact. Different letters indicate significant differences

Parasitaemia of infected canaries increased after the first immune challenge (Table 1B, Fig. 2). There was no difference between the sexes (Table 1B).

**Fig. 3:**
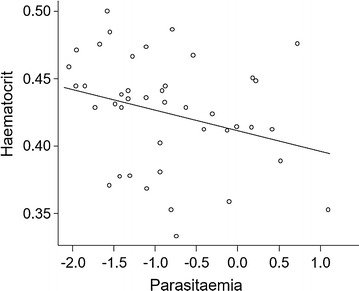
Haematocrit (%) as a function of parasitaemia (arbitrary unit, log transformed) in infected individuals

**Fig. 4:**
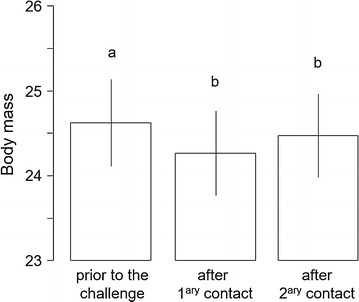
Body mass (g) as a function of antigen contact prior to and after immune challenges. Different letters indicate significant differences

Haematocrit was not affected by the immune challenge, the infection status, the sex or any interaction between these factors (Table 1C) but there was a negative association between haematocrit and parasitaemia (lme, t = − 2.55, p = 0.0171, Fig. 3). Body mass slightly decreased after the first immune challenge (Table 1D, Fig. 4). There was no difference of body mass between infection groups or between sexes (Table 1D).

Daily food consumption slightly increased over time after the first immune challenge and was negatively affected by the number of canary per cage (Table 1E).

## Discussion

To investigate the effect of *Plasmodium* infection on the ability of birds to mount an immune response to a new pathogen, a laboratory experiment was conducted by challenging birds with a novel non-pathogenic antigen and by measuring their antibody production in response to this challenge. *Plasmodium* infected individuals tended to have a lower immune response than uninfected ones, yet this difference was not statistically significant. However, parasitaemia increased after immune challenge, suggesting a cost of immune activation on the ability to control *Plasmodium* infection.

*Plasmodium* infected individuals were expected to have a weaker response to a novel antigen than uninfected ones, because *Plasmodium* parasites may drive host immuno-depletion [23] and because *Plasmodium* infection may impair host ability to invest in an energetically costly immune response [30]. The results showed no significant differences between uninfected and infected individuals. Mounting an immune response was however associated with an increased parasitaemia that negatively correlated with haematocrit. During chronic infection, a low parasite intensity could be maintained through host action via both antibody-dependent and independent mechanisms of acquired and innate immunity [31, 32]. Following a novel infection, immune effectors may change their target to the novel antigen, releasing host control on *Plasmodium* parasites and enabling the parasite to increase. Recrudescence, an increase of *Plasmodium* parasite intensity in the blood [33], has already been linked with host immunity in a mouse-*Plasmodium chabaudi* system, where a decrease in mouse immune functions was associated with *Plasmodium* recrudescence [34]. The result suggests the existence of a trade-off between immune control of chronic infection and immune response to a new pathogen, which might be common in nature where individuals often incur multiple infections at once.

Mounting an immune response has been shown to be energetically and metabolically costly [6, 7, 9, 10]. Here, following the first immune challenge, body mass slightly decreased and food consumption increased in all groups suggesting an energetic cost of mounting an immune response.

In the present study, the immune response did not differ between females and males. This is in contrast with previous hypothesis about sex differences in immune functions [35], thought to be mediated, for instance, by hormonal differences between the two sexes [36–38]. Canaries however, are not a sexually dimorphic species, individuals were not reproducing during the experiment and sexes were maintained separately in aviaries. In this experiment, therefore, males did not need to invest in costly reproductive behaviour or signals, which may explain a similar immune investment in both sexes.

In the present study, *P. relictum* (lineage SGS1) was used to infect birds. *P. relictum* is a species exhibiting genetic diversity and to a larger extent *Plasmodium* is a diverse group of parasites [33]. Yet, virulence is the result of the interaction of the parasite genotype, the host genotype and the environment the association occurs in [39, 40]. Therefore, pathogenicity (measured as parasitaemia) have been shown to vary depending on the species-specific association [41]. Thus, different levels of immune response might also be observed depending on the host and parasite species involved in the interaction under study.

## Conclusions

The short-term costs of mounting an immune response, such as the energetic demand, are usually considered to be low [42]. In contrast, the long-term costs, through immunopathology and cumulated oxidative damage, are likely to be more important [11, 42]. In the present study, the results showed that one short-term cost of mounting an immune response may be the released host control on parasite intensity during the chronic phase of *Plasmodium* infection. This may have limited costs (in terms of anaemia and weight loss) for individuals maintained in the laboratory with sufficient food resources and minimal stress. However, costs could be much higher for wild individuals, which have to deal with more stressful conditions such as food shortages or a harsh environment. The costs may also be paid later in life if the recrudescence of *Plasmodium* parasites is associated with supplementary oxidative damage to the host. Further studies on the mechanisms involved in the control of chronic infections would allow a better understanding of the mechanisms underlying *Plasmodium* recrudescence during chronic infection, an important feature of the ecology of this parasite.

## Abbreviations

ELISA: enzyme-linked immunosorbent assay
KLH: keyhole limpet haemocyanin
PBS: phosphate buffered saline

## References

[1] Knowles SCL, Palinauskas V, Sheldon BC (2010). Chronic malaria infections increase family inequalities and reduce parental fitness: experimental evidence from a wild bird population. J Evol Biol.

[2] Atkinson CT, Saili KS, Utzurrum RB, Jarvi SI (2013). Experimental evidence for evolved tolerance to avian malaria in a wild population of low elevation Hawai’i’Amakihi (*Hemignathus virens*). EcoHealth.

[3] Asghar M, Hasselquist D, Hansson B, Zehtindjiev P, Westerdahl H, Bensch S (2015). Hidden costs of infection: chronic malaria accelerates telomere degradation and senescence in wild birds. Science.

[4] Sheldon BC, Verhulst S (1996). Ecological immunology: costly parasite defences and trade-offs in evolutionary ecology. Trends Ecol Evol.

[5] Norris K, Evans MR (2000). Ecological immunology: life history trade-offs and immune defense in birds. Behav Ecol.

[6] Demas GE, Drazen DL, Nelson RJ (2003). Reductions in total body fat decrease humoral immunity. Proc Biol Sci.

[7] Demas GE (2004). The energetics of immunity: a neuroendocrine link between energy balance and immune function. Horm Behav.

[8] Demas GE, Chefer V, Talan MI, Nelson RJ (1997). Metabolic costs of mounting an antigen-stimulated immune response in adult and aged C57BL/6J mice. Am J Physiol.

[9] Eraud C, Duriez O, Chastel O, Faivre B (2005). The energetic cost of humoral immunity in the Collared Dove, *Streptopelia decaocto*: is the magnitude sufficient to force energy-based trade-offs?. Funct Ecol.

[10] Martin LB, Scheuerlein A, Wikelski M (2003). Immune activity elevates energy expenditure of house sparrows: a link between direct and indirect costs?. Proc Biol Sci.

[11] Sorci G, Faivre B (2009). Inflammation and oxidative stress in vertebrate host-parasite systems. Philos Trans R Soc Lond B Biol Sci.

[12] Costantini D, Møller AP (2009). Does immune response cause oxidative stress in birds? A meta-analysis. Comp Biochem Physiol A: Mol Integr Physiol.

[13] Sell S (2001). Immunology, immunopathology and immunity.

[14] Bertrand S, Criscuolo F, Faivre B, Sorci G (2006). Immune activation increases susceptibility to oxidative tissue damage in Zebra Finches. Funct Ecol.

[15] Alonso-Alvarez C, Bertrand S, Devevey G, Prost J, Faivre B, Chastel O (2006). An experimental manipulation of life-history trajectories and resistance to oxidative stress. Evolution.

[16] Hanssen SA, Hasselquist D, Folstad I, Erikstad KE (2004). Costs of immunity: immune responsiveness reduces survival in a vertebrate. Proc Biol Sci.

[17] Schmid-Hempel P (2008). Parasite immune evasion: a momentous molecular war. Trends Ecol Evol.

[18] Sacks D, Sher A (2002). Evasion of innate immunity by parasitic protozoa. Nat Immunol.

[19] Zambrano-Villa S, Rosales-Borjas D, Carrero JC, Ortiz-Ortiz L (2002). How protozoan parasites evade the immune response. Trends Parasitol.

[20] Finney OC, Riley EM, Walther M (2010). Regulatory T cells in malaria—friend or foe?. Trends Immunol.

[21] Hansen DS, Schofield L (2010). Natural regulatory T cells in malaria: host or parasite allies?. PLoS Pathog.

[22] Crompton PD, Moebius J, Portugal S, Waisberg M, Hart G, Garver LS (2014). Malaria immunity in man and mosquito: insights into unsolved mysteries of a deadly infectious disease. Annu Rev Immunol.

[23] Hisaeda H, Yasutomo K, Himeno K (2005). Malaria: immune evasion by parasites. Int J Biochem Cell Biol.

[24] Cellier-Holzem E, Esparza-Salas R, Garnier S, Sorci G (2010). Effect of repeated exposure to *Plasmodium relictum* (lineage SGS1) on infection dynamics in domestic canaries. Int J Parasitol.

[25] Hasselquist D, Wasson MF, Winkler DW (2001). Humoral immunocompetence correlates with date of egg-laying and reflects work load in female tree swallows. Behav Ecol.

[26] Jenkins T, Delhaye J, Christe P (2015). Testing local adaptation in a natural great tit-malaria system: an experimental approach. PLoS ONE.

[27] Hellgren O, Waldenström J, Bensch S (2004). A new PCR assay for simultaneus studies of *Leucocytozoon*, *Plasmodium*, and *Haemoproteus* from avian blood. J Parasitol.

[28] R Development Core Team. R: a language and environment for statistical computing. R Found. Stat. Comput. R Foundation for Statistical Computing; 2011.

[29] Crawley MJ (2007). The R book.

[30] Lochmiller RL, Deerenberg C (2000). Trade-offs in evolutionary immunology: just what is the cost of immunity?. Oikos.

[31] Stevenson MM, Riley EM (2004). Innate immunity to malaria. Nat Rev Immunol.

[32] Taylor-Robinson AW (2010). Regulation of immunity to *Plasmodium*: implications from mouse models for blood stage malaria vaccine design. Exp Parasitol.

[33] Valkiunas G (2005). Avian malaria parasites and other haemosporidia.

[34] McLean SA, Pearson CD, Phillips RS (1982). *Plasmodium chabaud*i: relationship between the occurrence of recrudescent parasitaemias in mice and the effective levels of acquired immunity. Exp Parasitol.

[35] Hasselquist D (2007). Comparative immunoecology in birds: hypotheses and tests. J Ornithol.

[36] Klein SL (2004). Hormonal and immunological mechanisms mediating sex differences in parasite infection. Parasite Immunol.

[37] Klein SL (2000). The effects of hormones on sex differences in infection: from genes to behavior. Neurosci Biobehav Rev.

[38] Folstad I, Karter J (1992). Parasites, bright males, and the immunocompetence handicap. Am Nat.

[39] Vale PF, Salvaudon L, Kaltz O, Fellous S (2008). The role of the environment in the evolutionary ecology of host parasite interactions. Infect Genet Evol..

[40] Wolinska J, King KC (2009). Environment can alter selection in host-parasite interactions. Trends Parasitol.

[41] Dimitrov D, Palinauskas V, Iezhova TA, Bernotienė R, Ilgūnas M, Bukauskaitė D (2015). *Plasmodium* spp.: an experimental study on vertebrate host susceptibility to avian malaria. Exp Parasitol.

[42] Hasselquist D, Nilsson J-Å (2012). Physiological mechanisms mediating costs of immune responses: what can we learn from studies of birds?. Anim Behav.

